# Evaluation of chirality descriptors derived from SMILES heteroencoders

**DOI:** 10.1186/s13321-025-01080-7

**Published:** 2025-08-31

**Authors:** Natalia Baimacheva, Xinyue Gao, Joao Aires-de-Sousa

**Affiliations:** 1 Faculty of Chemistry, University of Strasbourg, 4, Blaise Pascal Str., 67081 Strasbourg, France; 2 Faculty of Sciences, Université Paris Cité, 75013 Paris, France; 3 LAQV and REQUIMTE, Chemistry Department, NOVA School of Science and Technology, Universidade Nova de Lisboa, 2829-516 Caparica, Portugal

**Keywords:** Chirality, Neural network, Chiral HPLC, Artificial intelligence, Quantitative structure-enantioselectivity relationships, Random forest

## Abstract

Molecular representations of chirality, derived from latent space vectors (LSVs) of SMILES heteroencoders, were explored to train machine learning models to predict chiral properties, and were compared to conventional circular fingerprints. Latent space arithmetic was applied to enhance the representation of chirality, by calculating differences between the original descriptor of a molecule and the descriptor of its enantiomer, or the difference between the original descriptor and the descriptor obtained with the stereochemistry-depleted SMILES string. Machine learning was performed with the Random Forest algorithm applied to a dataset of 3858 molecules extracted from the literature (1929 pairs of enantiomers) to predict the elution order observed on the Chiralpak® AD-H column, as well as intrinsic structural chirality labels (R/S or canonical SMILES @/@@). The descriptors derived from the heteroencoders achieved an accuracy of up to 0.75 in the prediction of the elution order, and the fingerprints were superior (0.82). A better predictive ability was observed with the difference LSV descriptors than with the original descriptors.

**Supplementary Information:**

The online version contains supplementary material available at 10.1186/s13321-025-01080-7.

## Scientific contribution

Our work proposes latent space arithmetic to obtain descriptors of molecular chirality from SMILES heteroencoders. We used this molecular representation to build quantitative structure-enantioselectivity relationships for the prediction of the elution order of enantiomers in chiral chromatography and compared the results with those of circular fingerprints. We showed that delta descriptors of opposite enantiomers enhance the ability of latent space vectors to encode chirality.

**Supplementary Information:**

The online version contains supplementary material available at 10.1186/s13321-025-01080-7.

## Introduction

The application of neural network natural language models (NLMs) to the processing of molecular structures has been enabled by molecular representations based on linear notations such as SMILES strings (Simplified Molecular Input Line Entry System), fragSMILES [1], or SELFIES (SELF-referencIng Embedded Strings) [2]. fragSMILES were recently developed for *de novo* design with NLMs and allow to explicitly account for the chiral centers by incorporating their R/S absolute configuration. Neural networks of different types and architectures have been proposed not only for the automatic generation of molecules and reactions, but also for the derivation of molecular descriptors from latent space representations. Mizuno and co-workers [3] studied how a Transformer model learns chemical structures and partial chemical structures and observed that particularly long training is required to learn chirality.

We have previously demonstrated that delta latent space vectors (DLSVs) obtained from the original SMILES of the whole molecule and the SMILES of the same molecule with a target atom replaced can be used as atomic descriptors in Quantitative Structure–Property Relationships (QSPR), e.g. for the prediction of NMR chemical shifts [4]. DLSVs were also successful as molecular operators in the latent space, to transform molecules via halogenation reactions.

A similar approach is here described to explore new descriptors of molecular chirality (arising from chiral atoms), which are extracted from the latent space of SMILES heteroencoders. These neural networks process the whole SMILES strings. Therefore, the latent space representation includes the stereochemical configuration of chiral atoms, specified in the SMILES notation by the @/@@ symbols. Such latent space vectors are in themselves chiral descriptors that can in principle discriminate between opposite enantiomers. However, as the @/@@ symbols are typically a very tiny fraction of the string, it is expected that LSVs for opposite enantiomers are very similar, and machine learning of chiral properties from such descriptors may be difficult. The application of latent space arithmetic is proposed here to overcome this limitation and improve the ability of LSV descriptors of chirality to build successful QSPR/QSAR models for chirality-sensitive observable molecular properties.

Two alternative operations were investigated to emphasize the stereochemical information embedded in the latent space representation: (i) the difference between the LSV of a chiral molecule and its enantiomer (“ori-opp”), and (ii) the difference between the LSV of a chiral molecule and the LSV obtained from the same SMILES without stereochemistry labels (“ori-ns”). These approaches echo definitions of “chirality measures” developed more than 30 years ago [5].

Although thousands of molecular descriptors have been implemented in easily accessible software packages, only a few include chirality, i.e., can distinguish between enantiomers [6, 7]. Examples of developments from the last 20 years include chirality-sensitive flexibility descriptors for 3 + 3D-QSAR [8], TOMOCOMD-CARDD descriptors providing central chirality codification [9], enantioselective molecular asymmetry descriptors (EMAS) [10], simplex representation of molecular structures [11] and word embedding algorithms [12]. Morgan circular fingerprints encode the presence of atom-centered substructures and incorporate stereochemical tags (typically the Cahn-Ingold-Prelog labels, CIP) [13, 14]. Different from the molecular descriptors-based approach, derivatives of graph neural networks have been proposed that load molecular structures directly as graphs and incorporate 3D features such as bond lengths, bond angles and chiral tags for labeling the handedness of chiral centers [15], or torsion angles [16]; applications include the prediction of optical rotation [16], ligand binding affinity [16] and the prediction of retention time in chiral chromatography [15].

Chromatography on chiral stationary phases can separate enantiomers. The prediction of the chromatographic elution order of enantiomers from their molecular structures can support the assignment of absolute configurations [17] and assist in the automatic validation of experimental data. Chirality codes derived from 2D or 3D structures were used to train QSPR models to predict the elution order in a chiral high-performance liquid chromatography (HPLC) column [18]. Rio and Gasteiger developed chiral enantiophore descriptors from atoms of specific atom types and their distances to the chiral center and applied them to predict the elution order of enantiomers in liquid chromatography [19]. Natalini et al. [20] derived molecular descriptors from calculated 3D complexes between a chiral additive and analytes to predict the elution order of enantiomers in chiral ligand-exchange chromatography (CLEC) systems operating in the presence of a chiral mobile phase (CMP). De Gauquier et al. [21] calculated chiral descriptors based on scalar triple products for 3D molecular geometries and combined them with achiral descriptors to train MLR and PLS models and predict chromatographic behavior including the elution order of enantiomers in chiral HPLC columns. Liu et al. [22] proposed CatBoost models trained with 3D molecular descriptors to predict the retention times of chiral analytes enantioseparated by HPLC using cyclodextrin derivatives. Hong et al. [23] predicted chiral stationary phases to separate enantiomers (and their elution order) with deep neural networks trained with conformations of compounds represented by the x, y and z coordinates of atoms.

**Fig.1:**
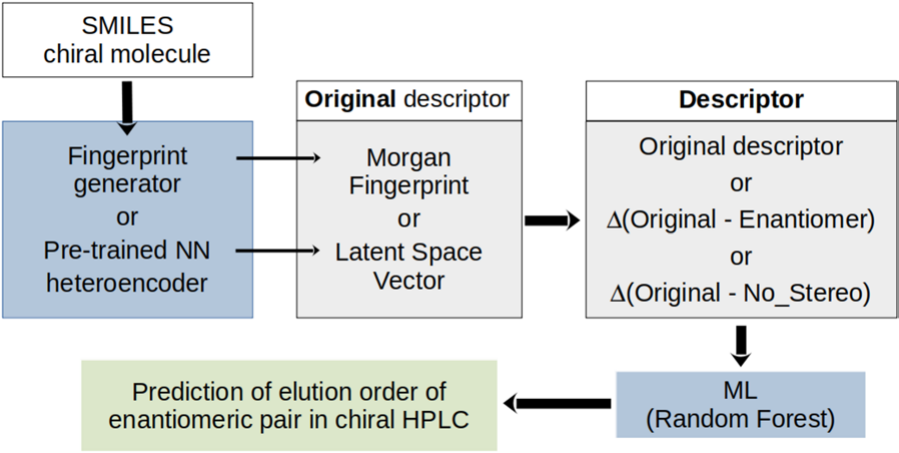
The procedure to transform chiral molecules into suitable chiral descriptors followed by machine learning (ML) to predict chiral properties. The original descriptors (fingerprints or latent space vectors) are used as such (“ori”), or as the difference between the original and that of the opposite enantiomer (“ori-opp”), or as the difference between the original and the descriptor for the stereo-depleted SMILES representation (“ori-ns”)

Here we explore the new chirality descriptors derived from the latent space of two SMILES heteroencoders trained to translate between SMILES strings and compare them with Morgan fingerprints encoding structural fragments (atomic circular neighborhoods). While chirality is included in SMILES with the “@”/“@@” label as a parity tag internal to the SMILES string, the Morgan fingerprints specify the stereochemistry of chiral centers with the CIP R/S labels. First, we investigated the ability of the LSV descriptors to predict the CIP label with the aim of testing their chemical significance in relation to an implicit property that can be derived from the molecular structure. We then challenged the new descriptors with the QSPR task of predicting the elution order of enantiomers observed on the Chiralpak AD-H column (a classification task with two classes, first eluted or last eluted enantiomer of the pair), compared with the results of fingerprints and inspected outliers. The procedure is illustrated in Fig. 1.

## Computational methods

### Data

The data were retrieved from the CMRT (chiral molecular retention time) dataset [15], which contains the retention times of 25,847 molecules (11,720 enantiomeric pairs) extracted from 644 articles. The dataset includes values for 25 types of HPLC columns with experimental conditions. The largest subset corresponds to the Chiralpak AD-H column with 5418 molecules, and we restricted our study to this subset. Some entries related to papers with inconsistent duplicates, unclear specifications of “major/minor” enantiomers, and molecules with more than one chiral center or elements other than H, B, C, N, O, F, P, S, Cl, Br, or I were discarded. The final dataset consisted of 3858 molecules (1929 pairs of enantiomers). Each enantiomer of a pair was classified as first or last eluted on the basis of the retention times obtained in the same experiment. Splitting into a training set and a test set (9:1 ratio) was performed randomly but both enantiomers of a pair were always in the training or test set, never separated. The final training set contained 1735 pairs of enantiomers, leaving 194 pairs for the test set. To compare the proposed models fairly, the choice of training and test entries was fixed and identical for all the models, except in the experiment with 5 alternative splits.

Experimental data found in literature and concerning the assignment of absolute configuration of enantiomers from chiral chromatography are prone to errors since separations are often performed with mixtures enriched on one enantiomer and not compared with a racemic mixture, and the absolute configuration can be assigned from various possible methods with different levels of uncertainty.

The molecular structures were retrieved from the CMRT database in the SMILES format. The RDKit software version 2024.09.1 was used to obtain canonical SMILES, as well as the CIP stereochemical labels (with the rdkit.Chem.rdCIPLabeler module).

### Molecular descriptors and chirality representation

Two open-source models were used to generate latent space descriptors: the Transformer [3] and the Continuous and Data-Driven Descriptors (CDDD) [24] models (available at https://github.com/mizuno-group/ChiralityMisunderstanding and https://github.com/jrwnter/cddd, respectively).

Among the various Transformer models published by Yoshikai and coworkers, we used the “no_stagnation” model to obtain latent space representations according to the documentation in the GitHub project (section “Featurization”). The model had been implemented with a dimension of 512, the dimension of the feed-forward layer was 2048, and the encoder and decoder had six layers; ReLU activation was used, and the dropout ratio was 0.1 in both the encoder and the decoder [3]. The “no_stagnation” model corresponds to a case (initialization seed) in which learning stagnation due to chirality features was not observed [3].

The latent space vectors (LSVs) calculated with the CDDD model were obtained as indicated in its GitHub project documentation (“SMILES embedding”, section “Inference Module”) with the *–no-preprocess* flag. The preprocessing step would have removed chirality. Since differentiation between enantiomers was the main objective of the study, the CDDD descriptors were obtained without preprocessing, from the canonical SMILES previously generated.

Morgan fingerprints were generated with the extended connectivity fingerprints algorithm [ 14] implemented by the RDKit library (2024.09.1). The GetMorganGenerator method of the rdFingerprintGenerator module was used for count fingerprints (with GetCountFingerprint). The fingerprints were generated with the following parameters: radius = 3 (number of iterations to grow the fingerprint), countSimulation = False, includeChirality = True (chirality information is added to the generated fingerprint), useBondTypes = True (bond types are included as a part of the default bond invariants), onlyNonzeroInvariants = False, includeRingMembership = True, countBounds = None (no boundaries for count simulation), fpSize = 512 (size of the generated fingerprint), and bondInvariantsGenerator = None, atomInvariantsGenerator = None. The CIP stereochemical labels were first obtained with the rdkit.Chem.rdCIPLabeler module. It was confirmed that the fingerprints were different for the two enantiomers of each pair in the data set.

In addition to the original (“ori”) descriptors (fingerprints or LSVs), two alternative delta descriptors were calculated for the 3 types of descriptors: a) the difference between the original descriptor of the molecule and of its enantiomer (“ori-opp") and b) the difference between the original descriptor of the molecule and of the descriptor obtained for the same SMILES without chirality (“ori-ns”). The ori-opp descriptors have opposite values for opposite enantiomers.

### Machine learning

#### TSNE

T-distributed stochastic neighbor embedding (t-SNE) [25] was used with the scikit-learn library [26] (version 1.5.1) via the sklearn.manifold.TSNE class to visualize the feature space of 3858 molecules represented by various possible types of descriptors. Maps were generated with the fingerprints or with the LSV descriptors using the original descriptors or the difference descriptors. After the calculation of the descriptors, each entry in our dataset also included the SMILES representations, the CIP label (R/S), the SMILES label (@/@@) and the order of elution (First/Last). The t-SNE models were fitted to the feature matrix with the following parameters: number of components = 2, learning rate = 200, PCA initialization, and perplexity = 30. The visualization of the t-SNE embedding in different features was enabled with the Plotly library (available at https://github.com/plotly/plotly.py) to create an interactive scatter plot, where each molecule was represented as a point with the t-SNE coordinates.

### Classification models

The classification models were trained for three endpoints: (a) absolute chiral configuration according to the CIP label (R/S); (b) number of “@” symbols in the canonical SMILES (@/@@); and (c) order of elution of the two enantiomers in the HPLC AD-H column (first/last). The first two endpoints aim at assessing the ability of the descriptors to identify implicit structural features (R/S labels for LSV and @/@@ for fingerprints) and explicit structural features (R/S labels for fingerprints and @/@@ for LSV).

The Random Forest (RF) algorithm [27] was used for classification. The models were implemented with the scikit-learn library [26] (version 1.5.1) via the sklearn.ensemble.RandomForestClassifier class using the following parameters: n_estimators = 100, bootstrap = True, oob_score = True, random_state = 0; all the additional parameters were set to default values.

### Outlier identification

For each prediction in the test set, prediction probabilities were retrieved. If each of the molecules in a pair of enantiomers was predicted with the incorrect class and the probability of each prediction exceeded 0.8, such cases were defined as outliers. Furthermore, the most similar molecule in the training set was identified for each outlier. Similarity was based on the Euclidean distance between the corresponding space vectors. The molecule at the shortest distance was considered to be the most similar.

## Results and discussion

### Ability of descriptors to identify structural features

**Fig.2:**
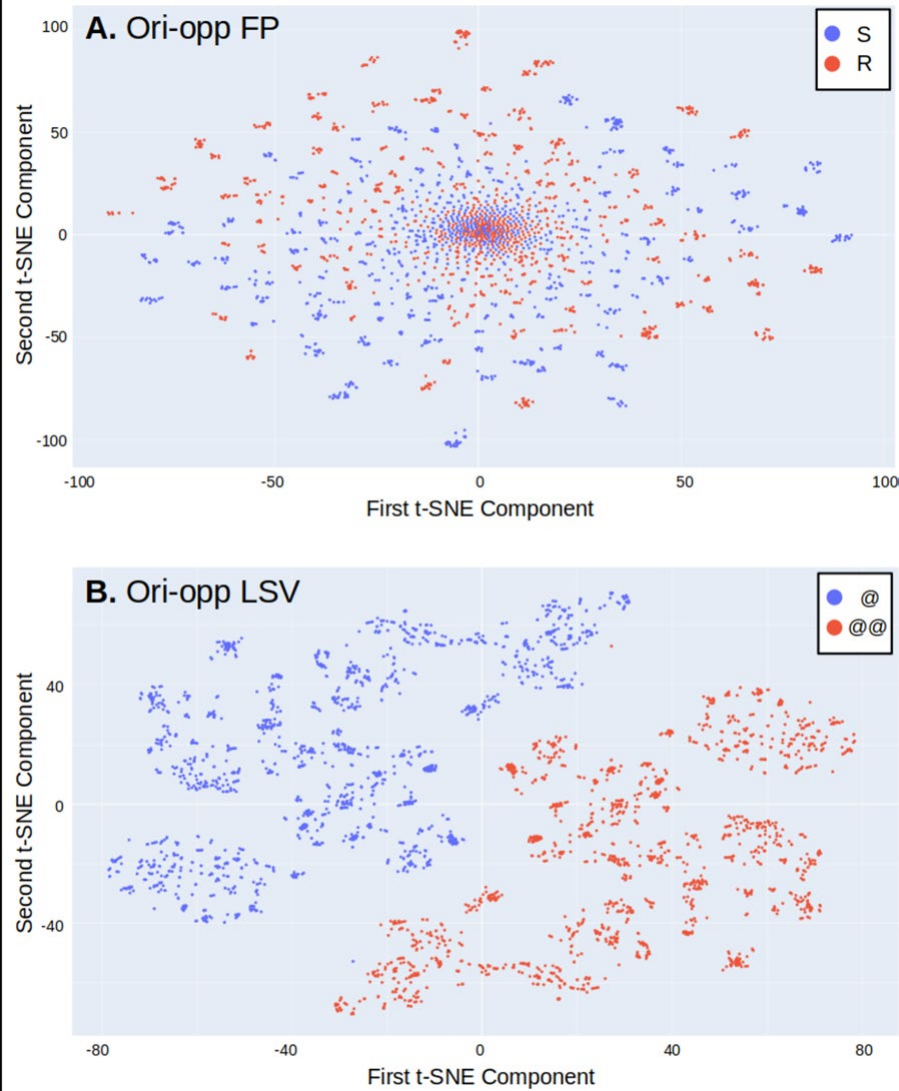
TSNE maps of the dataset using **A**: ori-opp fingerprint descriptors and CIP labels for colors (top map) and **B**: ori-opp Transformer LSV descriptors and SMILES @/@@ labels for colors (bottom map)

Two structural features are associated with the molecules in the data: @/@@ of the canonical SMILES and the R/S CIP labels. In our exploration of chirality descriptors derived from heteroencoders, we used the well-established Morgan fingerprints as a benchmark. The Morgan fingerprints encode substructures of the molecules, including the CIP labels of chiral atoms, but do not use the SMILES stereochemical labels. The heteroencoders directly use the SMILES representations with their stereochemical labels, but not their CIP labels. We first investigated the ability of the descriptors to identify the labels that were directly used for their generation (R/S for fingerprints, @/@@ for LSVs) and then their ability to identify the labels that were not used but that are implicit (R/S for LSVs and @/@@ for fingerprints). Experiments were performed with (a) the descriptors, (b) their difference to the opposite enantiomer (ori-opp) and (c) their difference to their stereo-depleted representation (ori-ns). TSNE unsupervised mapping and the random forest algorithm were used to explore the relationships between the descriptors and the stereochemical labels—Tables 1–2 and Fig. 2. In Tables 1–2 the out-of-bag (OOB) estimated accuracy is obtained from the aggregated predictions for the (randomly selected) objects that are left out of the training for each RF tree and are predicted by that tree.

**Table 1:** RF prediction of the CIP R/S label

Descriptor^a^	OOB accuracy^b^	Test set accuracy	% Correct pairs	% Undecided pairs
FP	0.802	0.912	84.0	14.4
FP ori-opp	0.922	0.954	94.3	2.0
FP ori-ns	0.901	0.925	87.1	10.8
Transf	0.575	0.817	72.2	19.1
Transf ori-opp	0.871	0.894	85.6	7.7
Transf ori-ns	0.814	0.902	86.6	7.2
CDDD	0.391	0.765	58.2	36.6
CDDD ori-opp	0.872	0.894	86.6	5.67
CDDD ori-ns	0.736	0.848	77.8	13.9

^a^*FP* Morgan fingerprint, *Transf* LSV from the Transformer model, *CDDD* LSV from the CDDD model, *ori-opp* difference between the descriptor of the molecule and of its enantiomer, *ori-ns* difference between the descriptor of the molecule and of its SMILES depleted of stereochemical information

^b^Global accuracy in the RF out-of-bag estimation with the training set

**Table 2:** RF prediction of the canonical SMILES stereochemical label (@ vs @@)

Descriptor^a^	OOB accuracy^b^	Test set accuracy	% Correct pairs	% Undecided pairs
FP	0.591	0.716	61.3	20.6
FP ori-opp	0.727	0.709	67.5	6.7
FP ori-ns	0.706	0.719	64.4	14.9
Transf	0.815	0.905	87.1	6.7
Transf ori-opp	0.999	1	100	0
Transf ori-ns	0.959	0.956	93.3	4.6
CDDD	0.712	0.92	84.0	16.0
CDDD ori-opp	1	1	100	0
CDDD ori-ns	0.955	0.987	97.4	2.6

^a^*FP* Morgan fingerprint, *Transf* LSV from the Transformer model, *CDDD* LSV from the CDDD model, *ori-opp* difference between the descriptor of the molecule and of its enantiomer, *ori-ns* difference between the descriptor of the molecule and of its SMILES depleted of stereochemical information

^b^Global accuracy in the RF out-of-bag estimation with the training set

The RF models trained with fingerprints could correctly identify the CIP label of up to 95% of the test set (Table 1) and the models trained with LSV could correctly identify all the SMILES labels of the test set (Table 2). Generally, the difference descriptors performed better than the original descriptors did, and the difference to the opposite descriptor was better than the difference to the nonchiral descriptor was. In line with the RF results, the Tt-SNE maps show that the SMILES stereo-label has a major impact on the delta LSV descriptors, whereas the CIP labels have a subtler effect on the profile of the delta fingerprints (Fig. 2). In the Transformer and the CDDD models the tokenization directly encodes ‘@’ or two consecutive ‘@’, which is clearly an advantage to learn these labels from the latent space vectors. Interestingly, the performance of the Transformer descriptors was comparable to that of the CDDD descriptors, which were derived from a heteroencoder trained with no stereochemical information. Although the CDDD model was trained with no stereochemical specification of the molecular structures, its vocabulary includes the “@” character. Therefore, the generated descriptors (LSVs) were different for the SMILES string representing a chiral molecule with one “@” character and for its enantiomer represented with “@@”.

**Fig.3:**
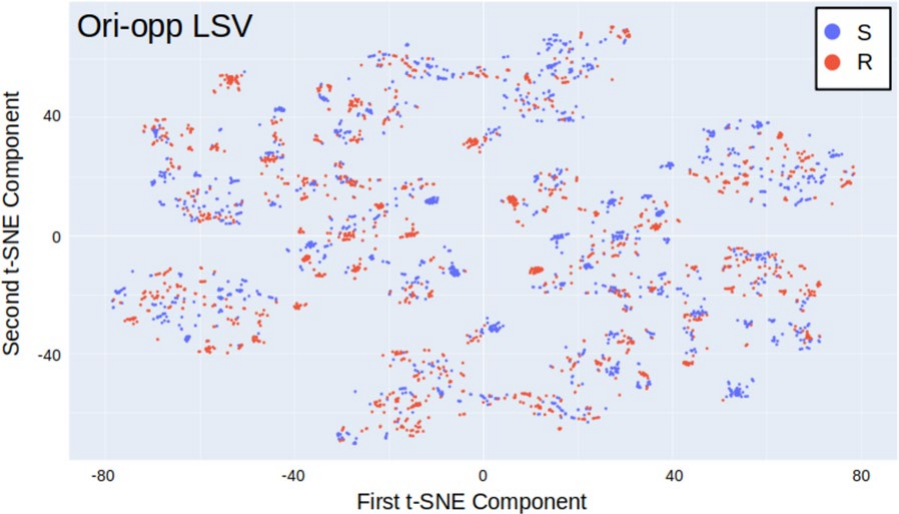
TSNE map of the dataset using the ori-opp Transformer LSV descriptors and the CIP labels for colours

With respect to the ability of the models to predict stereochemical labels that were not directly used for training, the models trained with LSVs could predict the CIP labels better than the models trained with fingerprints could predict the SMILES labels. There are typically groups of similar molecules in which one SMILES label is strongly associated with one CIP label (Fig. 3). This helps the models learn, making the induction of rules to predict labels from the descriptors easier.

The results in Tables 1–2 show that the OOB accuracy is lower than the accuracy for the test set in the experiments with the original descriptors. This is particularly significant in the prediction of the R/S labels with the Transformer and CDDD descriptors and in the prediction of the @/@@ labels with the fingerprints. This can be explained by the fact that the descriptors of the two enantiomers are globally very similar. If one enantiomer is in the training set, but not the other, the model is likely to predict the second as the first, which is wrong in terms of the stereochemistry label. While this cannot occur with the test set (made exclusively of pairs of enantiomers), it happens in the out-of-bag procedure in which a random selection of objects is left out of the training and is subsequently predicted to calculate the OOB error estimate. The OOB and test set accuracies are not very different in the experiments with the delta descriptors, which also shows the superior potential of delta descriptors to discriminate between enantiomers and to represent the chirality of molecules.

A further experiment was performed that supports the above explanation for the discrepancies between the OOB and the test set accuracies: one enantiomer of each pair in the test set was moved to the training set. It was observed that the OOB accuracies were not much changed, but the test set accuracies decreased to become similar to the OOB accuracies. The results were included in the Supplementary Information (Additional_file.xlsx).

### Ability of descriptors to predict the chromatographic elution order

The above-described experiments demonstrated that LSV descriptors (particularly delta LSV descriptors) encode a meaningful representation of molecular chirality. Next, we explored their ability to predict the elution order of a pair of enantiomers on Chiralpak AD-H column. Experiments were performed with the descriptors derived from the CDDD and the Transformer heteroencoders, and they were compared with the results obtained with the Morgan fingerprints (Table 3).

**Table 3:** RF prediction of the elution order on Chiralpak AD-H column

Descriptor^a^	OOB accuracy^b^	Test set accuracy	% Correct pairs	% Undecided pairs
FP	0.679	0.807	72.7	16.0
FP ori-opp	0.793	0.822	79.9	4.6
FP ori-ns	0.765	0.802	72.7	14.9
Transf	0.450	0.729	58.2	29.4
Transf ori-opp	0.759	0.753	70.6	9.3
Transf ori-ns	0.699	0.729	66.5	12.9
CDDD	0.339	0.683	50	36.6
CDDD ori-opp	0.772	0.732	67.5	11.3
CDDD ori-ns	0.637	0.727	61.9	21.6

^a^*FP* Morgan fingerprint, *Transf* LSV from the Transformer model, *CDDD* LSV from the CDDD model, *ori-opp* difference between the descriptor of the molecule and of its enantiomer, *ori-ns* difference between the descriptor of the molecule and of its SMILES depleted of stereochemical information

^b^Global accuracy in the RF out-of-bag estimation with the training set

The best results with the LSV-based descriptors were obtained with the delta LSV for opposite enantiomers derived from the Transformer heteroencoder, which achieved 75% correct predictions for the test set. This compares with the 82% obtained with the delta descriptors based on the Morgan fingerprints. Here, again, the performance of the delta descriptors was superior to that of the original descriptors, particularly with the LSV descriptors, and the difference to the opposite enantiomer yielded better results than the difference to the stereo-depleted descriptors. Experiments were also performed with 50, 100, 200 and 300 RF trees. The results were included in the Supplementary Information (Additional_file.xlsx). We observed some improvements in the OOB accuracy with 100 trees comparing to 50 trees, but further increasing the number of trees provided marginal improvements and made the model larger.

The robustness of the models was also investigated with five alternative random splits of the whole dataset (1929 pairs of enantiomers) into the training and test sets, always in the same proportion (9:1 ratio, 1735 and 194 pairs, respectively) and always including both enantiomers of a pair either in the training or in the test set. For each alternative split, models were trained with the different types of fingerprints and LSV descriptors, for the three different endpoints, using the same hyperparameters as for the Tables 1–3. The accuracies obtained for the test set and for the RF out-of-bag estimation are in the Supplementary Information (Additional_file.xlsx, worksheet “5_Alternative_splits”), as well as the averages and the standard deviations calculated across the five experiments with the five alternative training/test splits. The results showed robustness concerning the alternative random compositions of the training/test sets, with a maximum standard deviation of the test set accuracy of 0.032. Comparing the average accuracies to those in Tables 1–3 the only significant difference is that the advantage of delta descriptors comparing to the original descriptors was not clear in the case of the fingerprints. Additionally, the performance of the LSV descriptors in the prediction of the elution order was closer to that of the fingerprints (0.78 vs 0.81).

The heteroencoders and fingerprint descriptors are different in two ways: the representation of the molecular structure (by LSV derived from SMILES strings, or by substructures encoded in bits), and the stereochemical labeling (@/@@ according to the SMILES rules in the LSV, or the CIP labels in the fingerprints). To assess the impact of the second factor (the stereochemical labeling scheme) experiments were performed with the heteroencoder descriptors generated such that the @/@@ labels of the SMILES string corresponded to the CIP label (@ for S and @@ for R). The accuracy of the predictions thus obtained for the elution order was not significantly different, showing that the structure representation is the major factor, and that different stereochemical labeling schemes can represent chirality by LSVs derived from SMILES-trained heteroencoders. The details are in the Supplementary Information (Additional_file.pdf, Table S1).

### Inspection of outliers in the chromatography models

**Fig.4:**
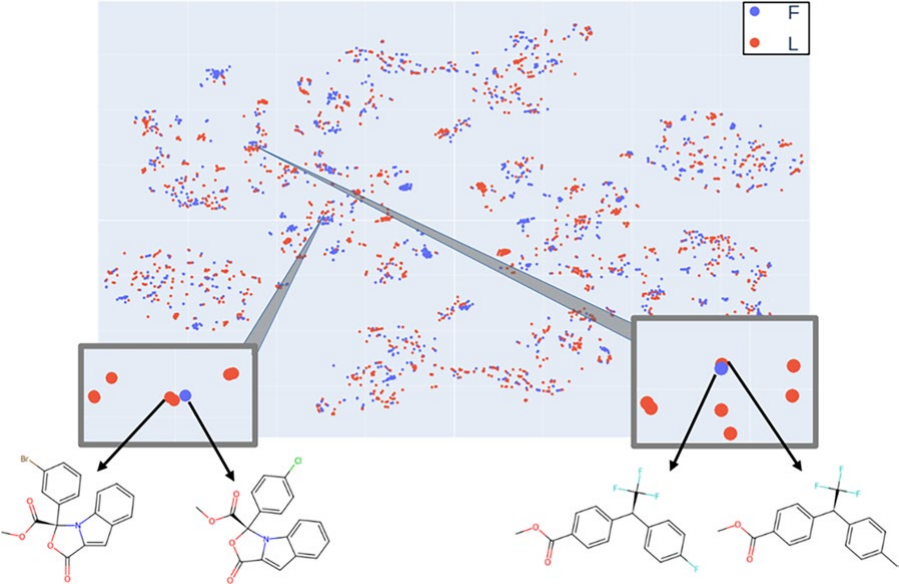
TSNE map of the dataset using ori-opp Transformer LSV descriptors (second t-SNE component vs. first t-SNE component). Two examples of outliers [28, 29] identified with the RF model are highlighted with their structures displayed, as well as the retrieved most similar molecules

**Fig.5:**
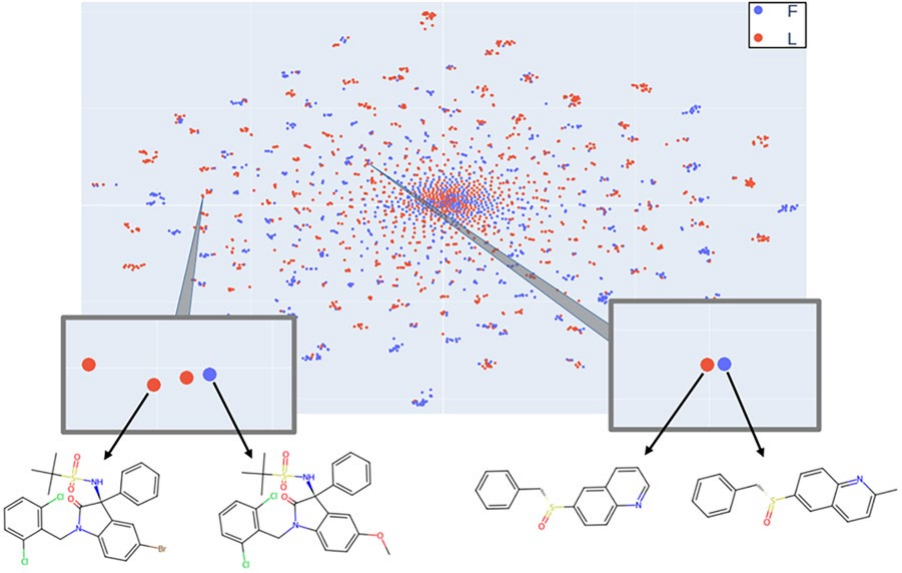
TSNE map of the dataset using ori-opp fingerprint descriptors (second t-SNE component vs. first t-SNE component). Two examples of outliers [30, 31] identified with the RF model are highlighted with their structures displayed, as well as the retrieved most similar molecules)

Test set outliers that were incorrectly predicted by the fingerprint- and Transformer-based best models with a high probability (RF probability > 0.8) were identified, and they were compared to their most similar molecules in the training set. They correspond to cases in which a very similar molecule exists in the training set, with a similar stereochemical configuration, but exhibit the opposite order of elution. The model predicts the same class for the test set molecule, which is wrong because they have experimental opposite chromatographic behaviors. Most of these molecules are aromatic and differ from their training set counterparts in one aromatic substituent, or in the nature of the aromatic ring. The t-SNE maps built with the same descriptors that were used for the RF models can also highlight the outliers. Figures 4 and 5 show examples of outliers identified by RF and the corresponding most similar training set counterparts on the same t-SNE maps. These cases illustrate “chirality cliffs” in which very similar molecules with similar stereochemical configurations (thus close to each other on the map) have opposite observable chiral properties (thus shown with different colors). They are obviously very difficult for models to learn and would require much larger training sets and/or completely different molecular representations. The two pairs of similar molecules in Fig. 4 are represented very similarly in canonical SMILES and differ only on their substituents: COC(= O)[C@]1(c2ccc(Cl)cc2)OC(= O)c2cc3ccccc3n21, COC(= O)[C@]1(c2cccc(Br)c2)OC(= O)c2cc3ccccc3n21, COC(= O)c1ccc([C@H](c2ccc(F)cc2)C(F)(F)F)cc1, COC(= O)c1ccc([C@H](c2ccc(C)cc2)C(F)(F)F)cc1.

## Conclusions

The incorporation of stereochemistry labels @/@@ in the SMILES representation of molecules enabled the LSV of heteroencoders to discriminate between enantiomers and provided descriptors that enabled ML algorithms to learn both intrinsic structural chiral features (CIP labels and canonical SMILES @/@@) and an experimentally observable property (elution order in chiral chromatography).

Although the descriptors derived from the heteroencoder trained with chirality information generally performed slightly better than those derived from the CDDD model (trained with no stereochemical information), the results were not very different.

The circular fingerprints were superior to the heteroencoders LSV descriptors in the prediction of the chromatographic elution order, but this difference was smaller in the experiment with the five random alternative training/test splits: while in Table 3 the best accuracy with a fingerprint is 0.82 and the best accuracy with a LSV descriptor (Transf ori-opp) is 0.75, the best average accuracy across the five experiments obtained with a fingerprint is 0.81 and with a LSV descriptor (CDDD ori-opp) is 0.78. However, the LSV-derived descriptors (generated with explicit @/@@ labels in SMILES) were better at predicting the CIP label (accuracy up to 0.90) than the fingerprints (incorporating CIP labels) at predicting the canonical @/@@ stereo identifiers (accuracy up to 0.72).

The results show that *delta* descriptors calculated with LSV enhanced the representation of chirality and enabled an improved ability to predict chiral properties.

The outliers of the chromatography model revealed cases in which small changes in the molecular structure (typically different aromatic rings) reversed the elution order.

## Supplementary Information

Supplementary Material 1.

Supplementary Material 2.

## Data Availability

The scripts for the random forest and t-SNE experiments, the data and the t-SNE maps are available from https://github.com/jairesdesousa/chiraldlsv. The CDDD model is available from https://github.com/jrwnter/cddd. The heteroencoder Transformer model is available from https://github.com/mizuno-group/ChiralityMisunderstanding.
