# Supplementary material for: Evaluation of chirality descriptors derived from SMILES heteroencoders
Source: J Cheminform. 2025 Aug 31;17:137. doi: 10.1186/s13321-025-01080-7 (PMC12398957; doi:10.1186/s13321-025-01080-7)
Supplement: Supplementary file 1 — Supplementary Material 1. [file 13321_2025_1080_MOESM1_ESM.pdf]

Supplementary Information

Evaluation of chirality descriptors derived from SMILES heteroencoders

Natalia Baimacheva, Xinyue Gao, Joao Aires-de-Sousa

Table S1: RF prediction of the elution order on Chiralpak AD-H column with the heteroencoder descriptors generated such that the @/@@ labels of the SMILES string corresponded to the CIP label (@ for S and @@ for R).

Descriptor[a]	OOB accuracy[b]	Test set accuracy	% Correct pairs	% Undecided pairs
Transf	0.471	0.678	52.1	31.4
Transf ori-opp	0.771	0.740	68.0	11.9
Transf ori-ns	0.716	0.711	63.9	14.4
CDDD	0.342	0.709	55.2	31.4
CDDD ori-opp	0.769	0.750	70.1	9.8
CDDD ori-ns	0.640	0.737	63.4	20.6

[a]FP: Morgan fingerprint, Transf: LSV from the Transformer model, CDDD: LSV from the CDDD model, ori-opp: difference between the descriptor of the molecule and of its enantiomer, ori-ns: difference between the descriptor of the molecule and of its SMILES depleted of stereochemical information; [b]Global accuracy in the RF out-of-bag estimation with the training set.

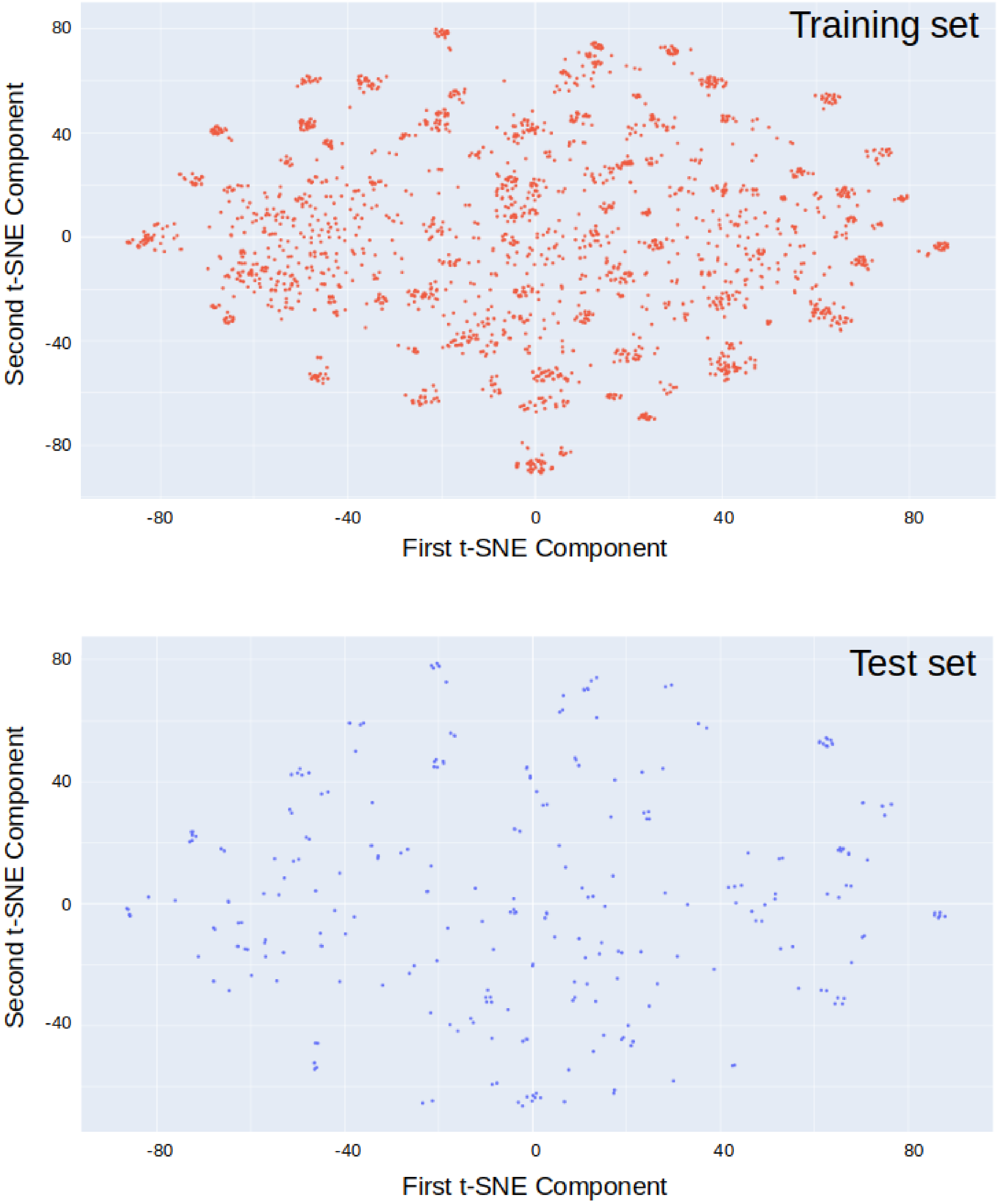

Figure S1: t-SNE map of the whole dataset using the fingerprint descriptors. The training and test sets are displayed separately in red and blue, respectively.

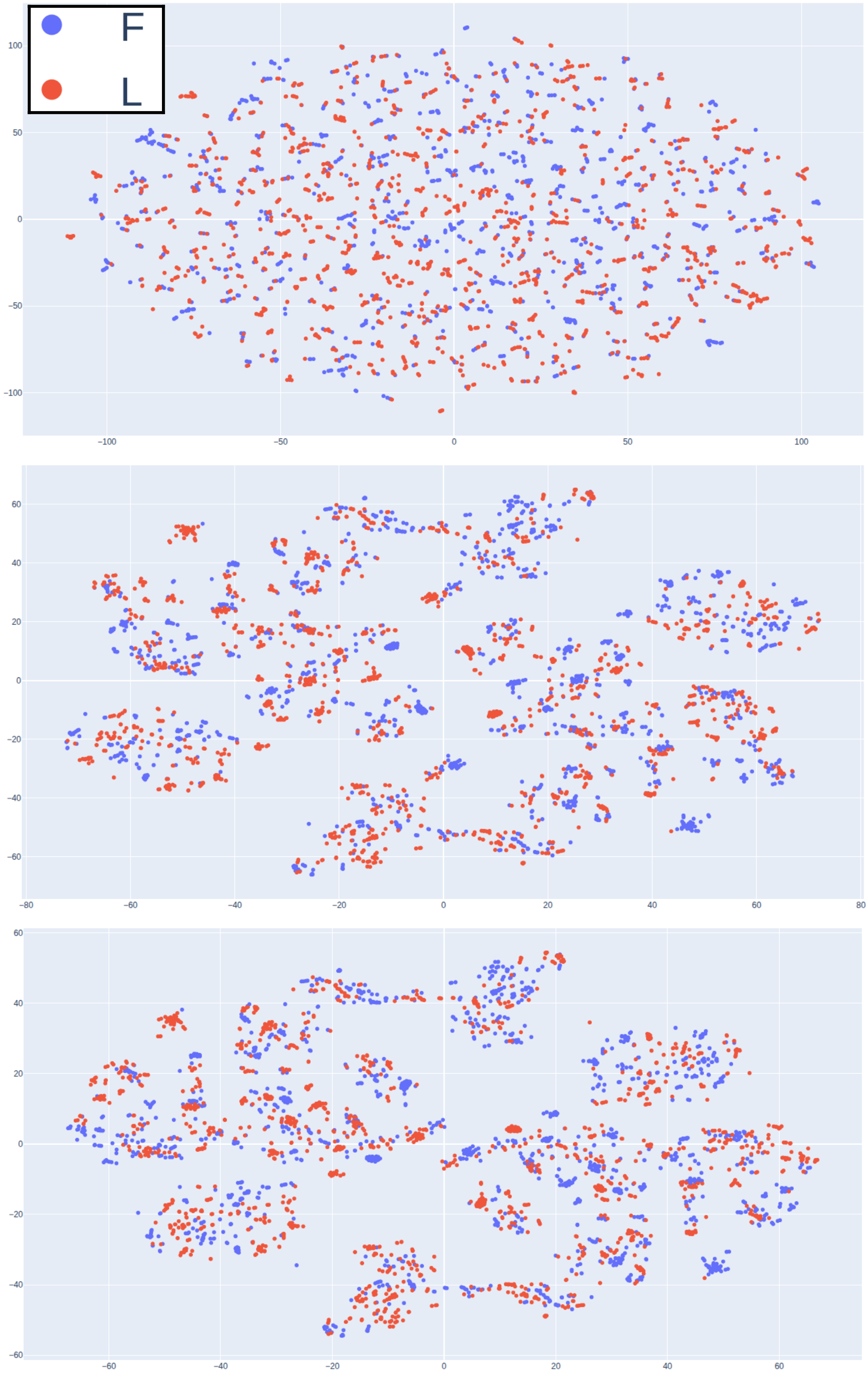

Figure S2: t.SNE map of the dataset using the ori-opp Transformer LSV descriptors (second t-SNE component vs. first t-SNE component) with learning rate = auto, max_iterations=1000 and **different perplexity values: 5 (top), 30 (center) and 50 (bottom)**.

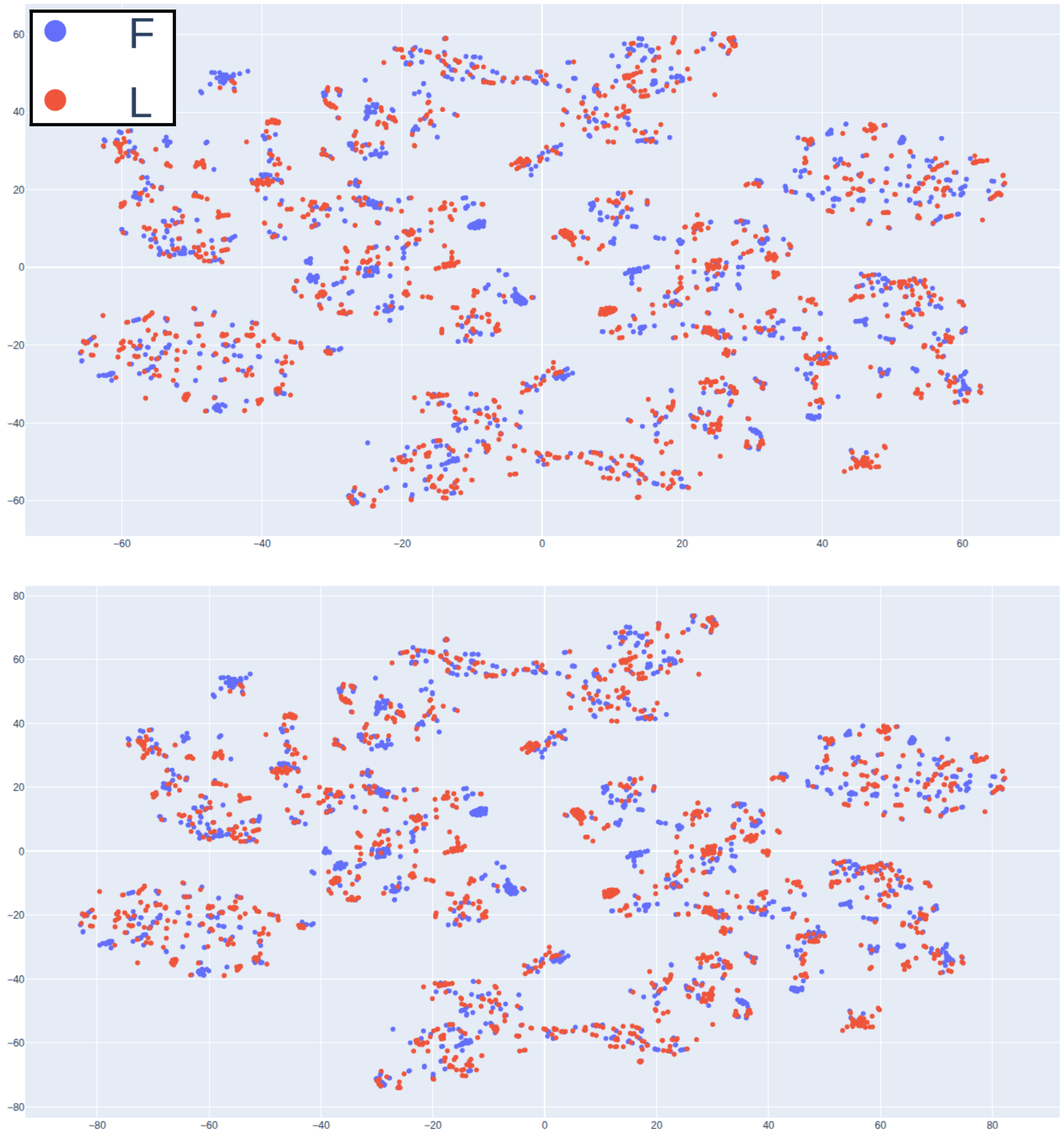

Figure S3: t-SNE map of the dataset using the ori-opp Transformer LSV descriptors (second t-SNE component vs. first t-SNE component) with perplexity = 30 and **different values of max_iterations and learning rate**. Top: max_iterations=1000 and learning rate = 50. Bottom: max_iterations=1500 and learning rate = auto.
